# Parameter Evolvability in Gene Expression Models Drives Phenotypic Adaptation

**DOI:** 10.1162/ISAL.a.869

**Published:** 2025-10-06

**Authors:** Pablo Japón, Jesús Miró-Bueno, Ángel Goñi-Moreno

**Affiliations:** 1 Systems Biology Department, Centro Nacional de Biotecnología (CSIC), Madrid, 28049, Spain

## Abstract

Gene expression transforms information encoded in DNA into functional protein activity. Although these systems are often assumed to be static—thus enabling the programming of genetic circuits to meet predefined specifications—they are, in fact, dynamic and subject to evolutionary change. As sequences accumulate subtle mutations, their phenotypic responses may shift, potentially undermining initial design goals. Here, we investigate the evolutionary distance between gene expression models and their future (mutated) versions over time, while tracing their phenotypic adaptation. We use four-parameter gene expression models as individuals and apply a genetic algorithm to evolve them towards both fixed and oscillatory protein targets. To quantify divergence in expression rates, we introduce a *parameter distance* metric that captures differences within and between individuals. When evolving towards a fixed phenotype, individuals often follow consistent patterns in distance-to-protein space (“V-shaped” trajectories), suggesting that certain parameter deviations enable faster adaptation. Furthermore, groups with identical phenotypic outputs but differing parameter distances follow distinct evolutionary paths to reach the same target. In fluctuating environments, large mutational steps across all parameters allow populations to closely track a moving protein target, while small steps result in the population being unable to keep pace with the target, leading to a delayed, repeated pattern in the protein response. These findings highlight that both the underlying parameter architecture—directly translatable into DNA sequences—and the scale of mutation critically shape adaptive dynamics. We advocate accounting for this effect to incorporate evolution as a design element in the engineering of biocomputations.

## Introduction

Living cells do more than passively store DNA; they continually process and transmit information through dynamic molecular networks. Transcription factors, signaling cascades, and feedback loops effectively constitute biochemical “channels” whose capacity and noise characteristics can be quantified much like engineered and computing systems (Tkačik and Walczak, 2016; Razo-Mejia et al., 2020; Amos and Goñi-Moreno, 2018). Crucially, these regulatory channels are themselves subject to mutation and selection: as organisms evolve, the very rules by which genetic information is read and interpreted can change (Adami, 2004; Wagner, 2005).

Engineered gene circuits Brophy and Voigt (2014); Tas et al. (2021a) often assume static behavior (i.e., DNA sequence or structure does not change due to evolution), yet evolutionary simulations reveal that even small networks can explore vast regions of functional space with minimal parameter adjustments (Mugler et al., 2008). Likewise, biological degeneracy—the ability of distinct molecular configurations to produce the same output—enhances both robustness and evolvability by providing multiple fallback routes under perturbation (Whitacre and Bender, 2010). Together, these findings reposition evolvability from a vague “side-effect” of life to a quantifiable property that can be deliberately assessed.

Synthetic biology has made great strides in assembling genetic parts to perform logic operations, regulate metabolism, and sense environmental cues (Tas et al., 2021b; Nielsen and Keasling, 2016). Yet even well-characterized circuits can degrade over generations as mutations accumulate in promoters, ribosome-binding sites, or coding sequences (Sleight et al., 2010). This tension between design stability and biological adaptability underscores the need for metrics that predict how engineered systems will drift—and how easily they might be steered back on course. Underlying this challenge is the idea of harnessing evolutionary dynamics to program living systems—an essential step toward realizing the full potential of cells as platforms for biocomputation Goñi-Moreno (2024).

In bacteria, gene expression dynamics at steady state can be captured by four rates: transcription (*K*_0_), translation (*K*_1_), mRNA degradation (*K*_2_), and protein degradation (*K*_3_) (Laursen et al., 2005; Mackie, 2013; Goldberg and St John, 1976). Multiple combinations of these rates produce the same protein level, meaning identical phenotypes can mask distinct “regulatory genotypes.” Quantifying divergence in this four-dimensional space is critical to understanding both natural evolution and the long-term behavior of synthetic circuits.

Phenotypic convergence—where different genetic changes yield similar traits—has been documented in evolutionary biology, from stickleback fish armor loss to microbial metabolic shifts (Rosenblum et al., 2014; Storz, 2016). At the molecular level, two cells with the same protein output can occupy very different points in rate-space, and these hidden differences may alter how quickly or by what pathway they adapt under new selection pressures (Hausser et al., 2019).

To investigate this hidden dimension of evolvability, we employ a genetic algorithm to evolve four-rate gene expression models toward fixed and oscillatory protein targets. We introduce a *parameter distance* metric to collapse four-dimensional divergence into a single scalar measure. We then ask: (i) Do populations with the same phenotype but different parameter distances follow distinct evolutionary routes? (ii) Does distance from the reference predict adaptation speed? (iii) How does the scale of mutational steps affect tracking of changing targets?

The remainder of the paper is organized as follows. First, we detail our gene-expression model, genetic algorithm, and parameter-distance definitions. Next, we present evolution toward fixed targets and characterize the characteristic “V-shaped” trajectories in distance–phenotype space and explore whether parameter distance influences evolutionary outcomes for populations starting at the same protein level. We then extend to dynamic, sinusoidal targets to examine the impact of mutational amplitude. Finally, we discuss how these insights into hidden regulatory variation inform both evolutionary theory and the future design of robust, evolvable biological circuits.

## Results

### Gene expression model and genetic algorithm

**Figure 1:**
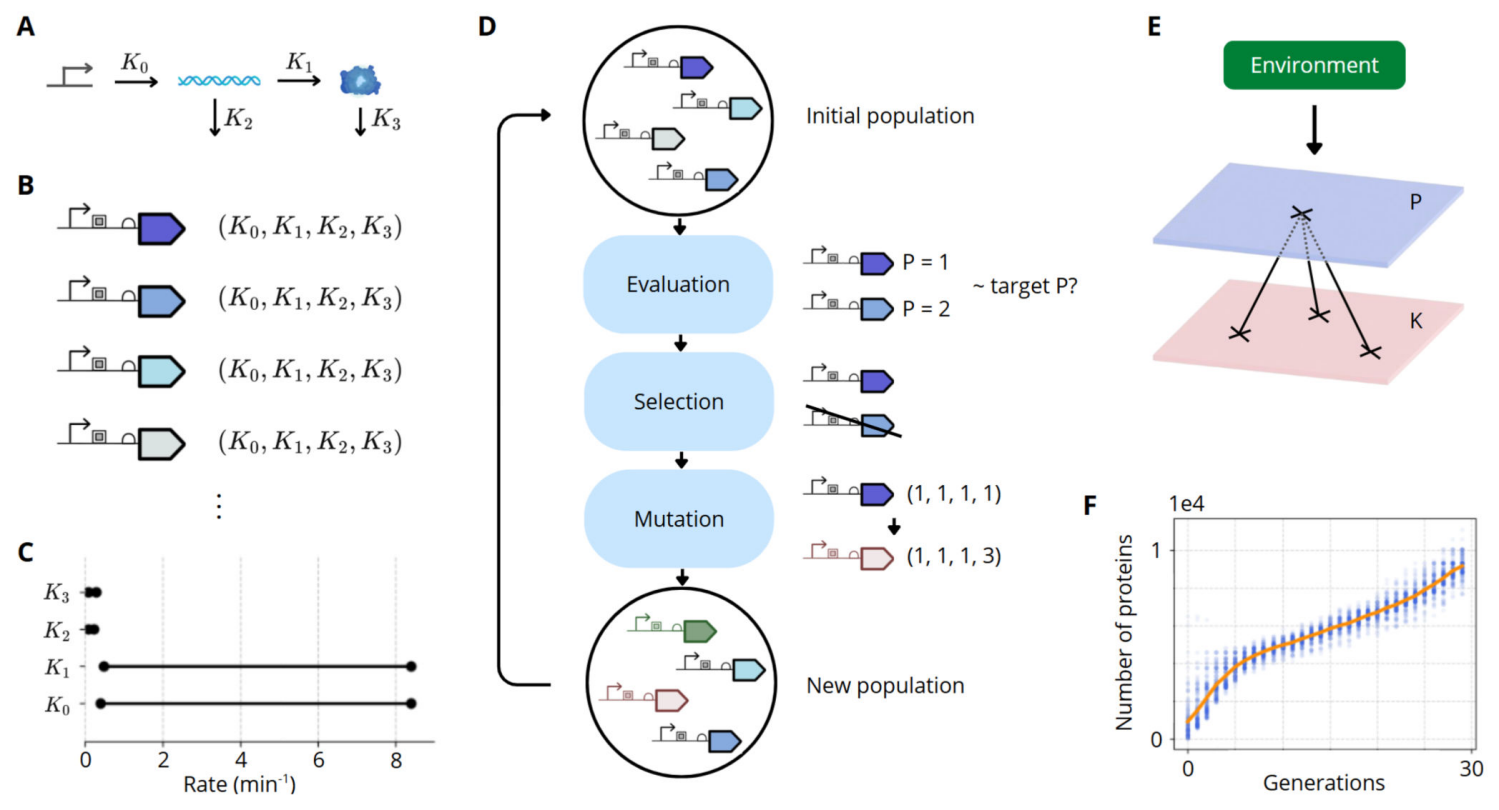
Gene expression model and genetic algorithm. **A)** Gene expression model defined by four key parameters: transcription rate (*K*_0_), translation rate (*K*_1_), mRNA degradation rate (*K*_2_), and protein degradation rate (*K*_3_). **B)** Each individual is identified by its unique set of these four parameters. **C)** Parameter ranges derived from literature (Bernstein et al., 2002; Nagar et al., 2021; Proshkin et al., 2010; Gong et al., 2008; Valleriani et al., 2011). **D)** Overview of the genetic algorithm process: an initial population with random parameters is evaluated based on its resulting protein level (phenotype). The fittest parameter sets—those producing protein levels closest to the target—are selected, and new offspring are generated through mutation. Over generations, the population shifts toward an optimal set of parameters, refining protein expression levels to meet the environmental target. **E)** The environment sets the target protein phenotype, defining the optimal expression level. The parameter space (K layer) at the bottom represents all possible combinations of (*K*_0_, *K*_1_, *K*_2_, *K*_3_), while the phenotypic space (P layer) at the top represents the corresponding protein output. These two layers are connected through the gene expression model, which dictates how parameter variations translate into phenotype differences. Selection in the phenotypic space leads to shifts in the underlying parameter space, shaping the evolutionary trajectory of the population. **F)** Simulation example showing evolutionary progression of a population across generations. Blue points represent all individual phenotypes in each generation, while the orange line indicates the population mean. The overall trend illustrates increasing protein output and convergence toward the target phenotype.

To study how gene expression parameters evolve under selection, we developed a model based on four key rates: transcription (*K*_0_), translation (*K*_1_), mRNA degradation (*K*_2_), and protein degradation (*K*_3_) (Figure 1A). At steady state, these rates determine the number of proteins according to:

(1) $$P_{\text{ss}}=\frac{K_{0}K_{1}}{K_{2}K_{3}}$$

Each individual in our simulation is uniquely characterized by a set of these four parameters that determine its protein production level (Figure 1B). Because multiple parameter sets can produce the same steady-state protein level, bacteria can share a phenotype yet differ substantially in their underlying rates. The values of these parameters were constrained by biologically relevant ranges obtained from the literature (Figure 1C).

We implemented a genetic algorithm to simulate the evolutionary adaptation of bacterial populations. The algorithm begins with an initial population of 200 individuals where each individual is assigned a random set of parameters (Figure 1D). These individuals are evaluated based on their protein expression levels, and those whose output is closest to an externally defined target phenotype are preferentially selected. Mutations introduce small variations in the parameters of selected individuals, leading to gradual shifts in the population’s composition. Over multiple generations, these shifts refine the parameter sets to optimize protein expression.

The relationship between parameter space and phenotypic space is critical in this process (Figure 1E). The K-layer represents all possible parameter combinations, while the P-layer reflects the corresponding protein expression levels. The gene expression model acts as the link between these spaces, determining how changes in parameters translate into phenotypic variation. Selection pressures applied in the phenotypic space drive shifts in the underlying parameter space, shaping the evolutionary trajectory of the population.

Figure 1F illustrates an example simulation, in which an initially random population undergoes selection and mutation over multiple generations. As expected, the mean protein expression level progressively increases, approaching the target phenotype, since bacteria with optimal parameter sets are favored by selection.

### Parameter distance as a measure of deviation from a reference set

To gain insight into how populations navigate parameter space, we introduce *parameter distance* — a metric that compares each parameter set to a reference set of values. Parameter distance helps us assess how far an individual’s gene-expression profile deviates from a predefined standard, providing a compact scalar summary of regulatory divergence without tracking each rate separately.

The parameter distance for each rate *K*_*i*_ is calculated as the relative deviation from the reference value *K*_*i*,ref_:

$$d_{K_{i}}=\frac{\left|K_{i}-K_{i,\;\text{ref}\;}\right|}{K_{i,\;\text{ref}\;}},$$

where *K*_*i*_ is the individual’s value for parameter *i*, and *K*_*i*,ref_ is the corresponding reference value. Summing over all parameters yields the total distance:

$$D=\sum_{i=0}^{3}d_{K_{i}}.$$

This approach mirrors how genetic and evolutionary distances aggregate differences across multiple loci or sites (Nei, 1972; Séré et al., 2017), providing a framework to analyze how “far” regulatory architectures lie from one another and how that distance influences evolutionary trajectories.

**Figure 2:**
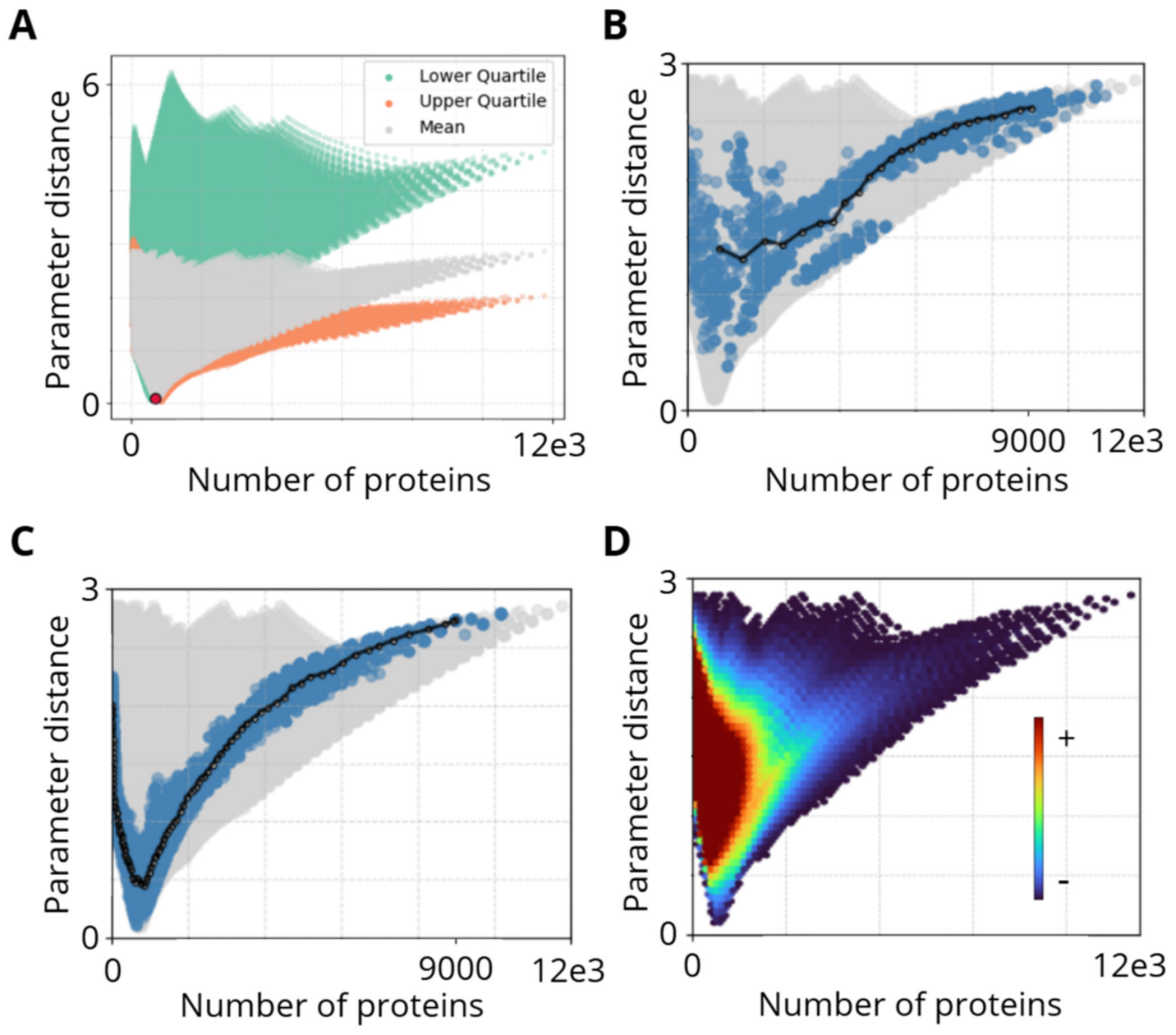
Reference parameters and V-shaped trajectories in parameter-protein space. **A)** Parameter distance (y-axis) versus protein output (x-axis) for all possible combinations of gene expression parameters, using three different reference parameter sets: lower quartile (green), midpoint (gray), and upper quartile (orange). The shaded regions illustrate the distribution of parameter distances relative to each reference set, highlighting how the choice of baseline influences the interpretation of parameter deviation. For this study, we adopt the midpoint values as the reference set, shown as a red dot in the plot. **B)** Evolution of a random population toward a fixed target protein output of 9000. Grey points represent all possible parameter combinations, blue points represent individual bacteria in the evolving population, and the black line shows the median trajectory over generations. **C)** Evolution in the opposite direction, from a homogeneous population with an initial protein output of 9000 toward a low-protein target of 10, showing a similar V-shaped trajectory, highlighting consistent evolutionary patterns in both directions. **D)** Density map of parameter distance vs. protein output, revealing regions of higher probability where evolutionary paths tend to converge. The high-density areas (red/yellow) represent parameter sets that appear more frequently, explaining why the trajectories in (B) and (C) consistently take a structured shape.

Figure 2A shows the distribution of parameter distance across all possible combinations of gene expression parameters, using three different reference sets. These represent low, mid-range, and high regimes within the biologically plausible parameter ranges.

As shown in Figure 2A, the distribution of parameter distances varies depending on the choice of reference set. The spread of the distances helps us understand how the position of the reference set influences the population’s distribution in the parameter space. Throughout the remainder of the study, we adopt the mid-range reference set as a biologically representative baseline for comparison.

Figure 2B and Figure 2C illustrate how parameter distance changes when populations evolve toward different target protein levels. In both cases, the population follows a structured V-shaped trajectory, indicating consistent patterns in how bacteria navigate parameter space during adaptation.

To understand why these trajectories take the shape they do, we computed a density map of the parameter distance–protein space (Figure 2D). The highest-density regions (red/yellow) correspond to parameter sets that are more frequently encountered in the space, indicating that certain parameter configurations are inherently more accessible.

This suggests that evolution does not proceed randomly through distance-protein space but instead follows structured pathways shaped by the density of viable solutions. Certain regions are easier to reach, while others are rarely visited, leading to the observed V-shaped evolutionary trends.

### Multiple pathways and lifetimes for the same initial phenotype

We next explore whether parameter distance influences evolutionary outcomes for populations starting at the same protein level. We tested two representative starting phenotypes—1,500 and 2,500 proteins—each subdivided into eight populations with distinct parameter distances. Our aim was to determine how these initial distances shape both the pathways taken in distance–protein space and the lifetimes (generations) required to reach a new target phenotype. Each population simulation was ran 500 times.

**Figure 3:**
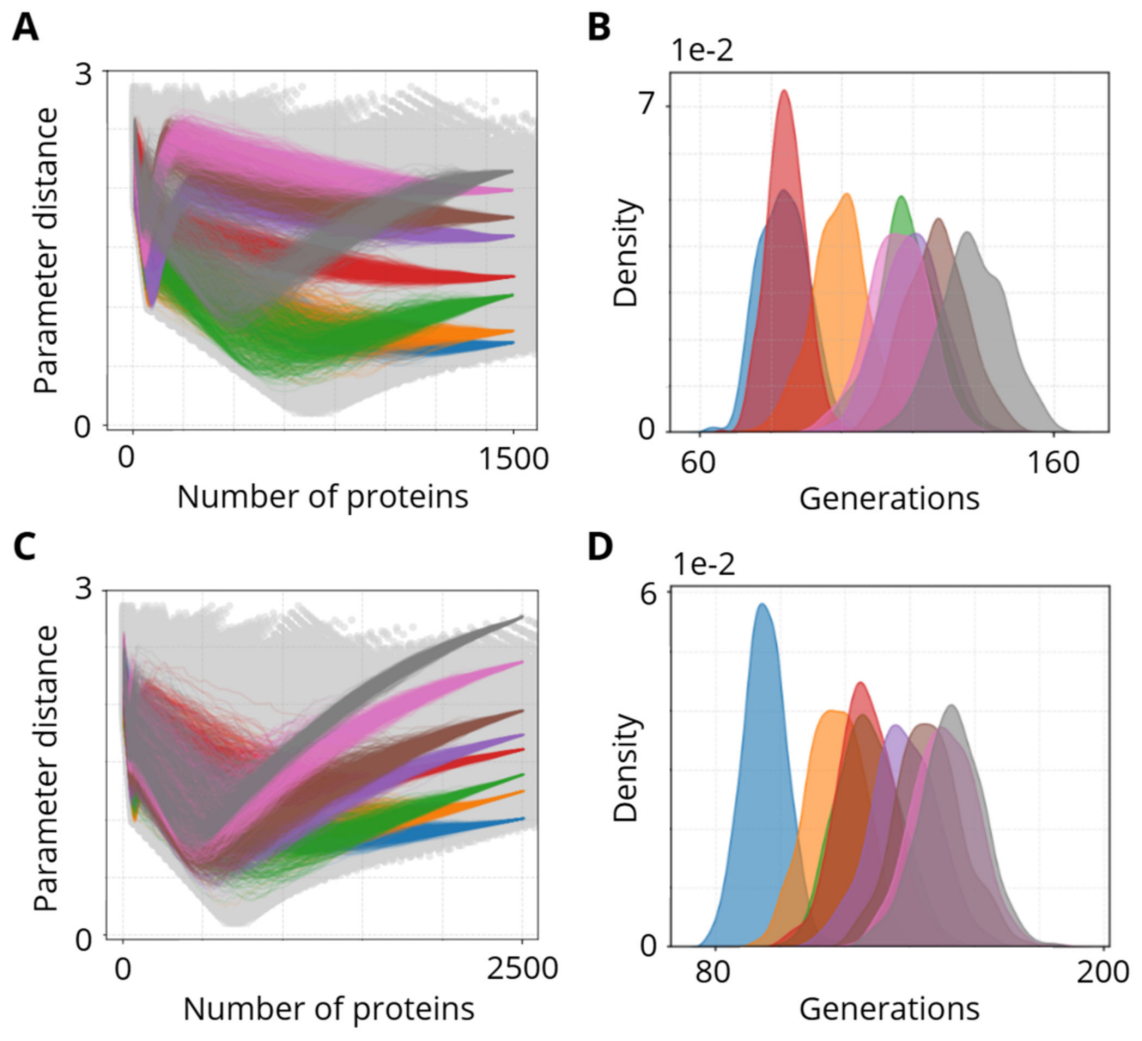
Variation in evolutionary pathways and life-times for populations sharing the same initial phenotype but differing in parameter distance. **A)** Overlaid trajectories in distance–protein space for eight populations all starting at 1,500 proteins, each with a distinct parameter distance from the reference set. Despite identical initial phenotypes, their evolutionary routes diverge considerably. **B)** Lifetime distributions (histograms or KDE plots) for the same populations, showing the number of generations required to reach the new target phenotype. Smaller initial distances often lead to faster convergence, though notable exceptions appear. **C), D)** Equivalent analyses for populations starting at 2,500 proteins. The patterns echo those in (a,b), indicating that parameter distance can strongly influence both the shape of the evolutionary path and the time to success, even when initial protein levels are held constant.

Figure 3A displays overlaid trajectories in distance–protein space for populations starting at 1,500 proteins. Despite identical initial phenotypes, the groups diverge into markedly different evolutionary “routes,” largely reflecting their parameter distance. Some follow a neat “V” shape and converge swiftly on the new target, whereas others meander more unpredictably. By contrast, the lifetime distributions in Figure 3B reveal how many generations each group needs to succeed, highlighting that closer-distance populations typically finish earlier, yet outliers arise in both directions. A similar pattern emerges for the 2,500-protein case (Figure 3C,D), underscoring that phenotype alone does not fully predict adaptation speed; the “hidden” dimension of parameter distance can accelerate or delay convergence.

Overall, these results confirm that even when two populations share the same starting phenotype, differences in how their gene expression parameters deviate from a reference set can drastically affect their evolutionary trajectories. While smaller distances often correlate with quicker adaptation, certain large-distance groups occasionally discover rapid alternative pathways. We do not attempt to explain these exceptions mechanistically, but they suggest that local constraints and stochastic factors in parameter space can yield surprising outcomes, emphasizing the nuanced role of parameter distance in shaping both evolutionary paths and timescales.

### Evolving under a dynamic, time-varying target

To explore adaptation in a fluctuating environment, we replaced the fixed protein target with a sinusoidally varying ideal phenotype. This scenario more closely mimics natural conditions where external factors oscillate rather than remain constant. We tested two mutation regimes: one permitting large changes in the translation rate (*K*_1_), and another restricting it to small increments.

In the first scenario, we allowed all four parameters (*K*_0_,*K*_1_,*K*_2_,*K*_3_) to mutate with large step sizes, providing maximal flexibility to respond to the changing environment.

**Figure 4:**
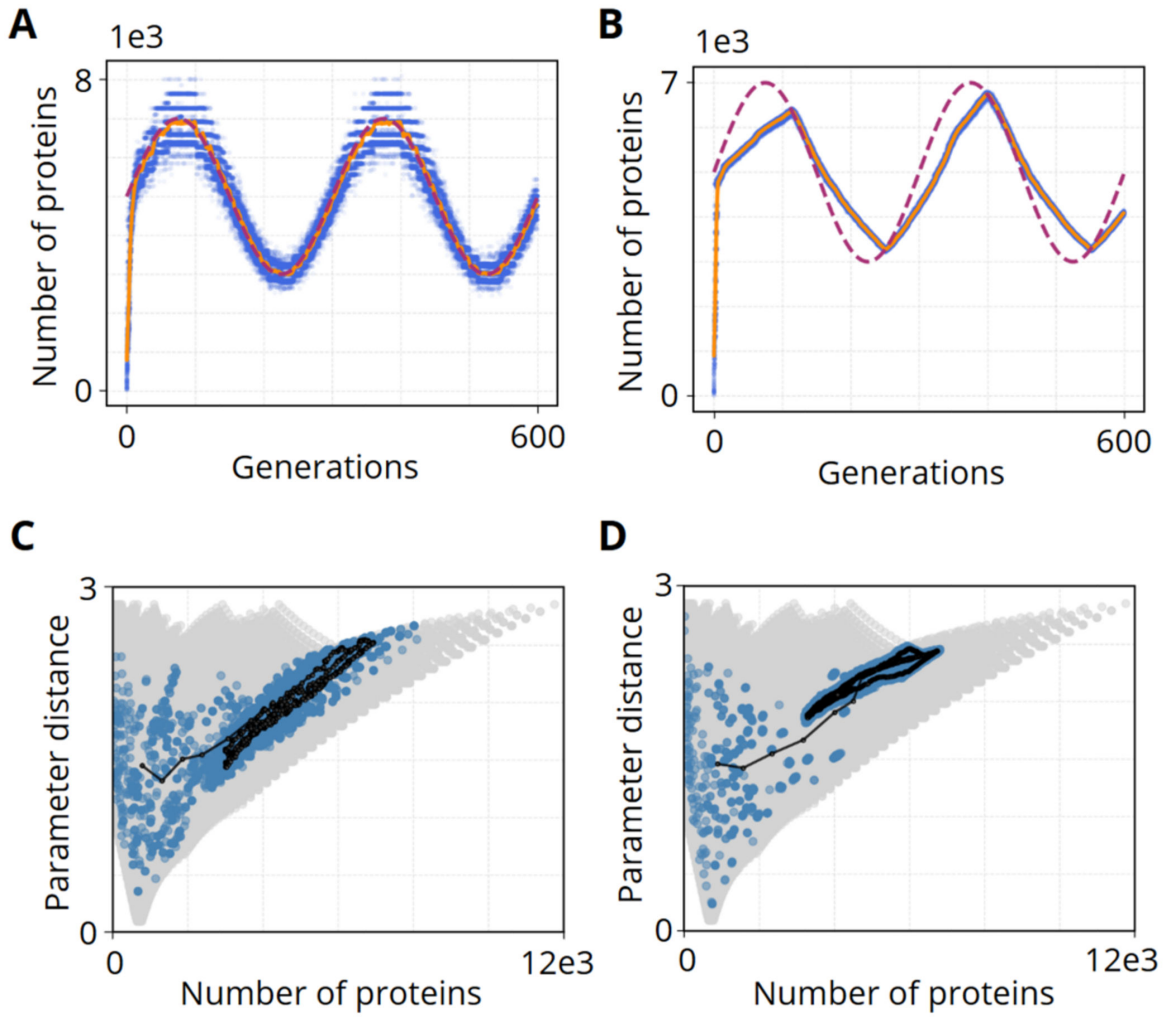
Adapting to a sinusoidally varying protein target under large vs. small parameter mutations. **A), C)** Large-step scenario: The left subplot shows the population’s mean protein output (orange) closely tracking the oscillatory target (red) across generations. The right subplot depicts trajectories in distance–protein space, where the population repeatedly shifts to match each peak and trough. **B), D)** Small-step scenario: The left subplot reveals a “sawtooth” pattern in protein output, reflecting the population’s lag behind rapid oscillations. The right subplot in distance–protein space confirms slower, more incremental paths, leading to less effective alignment with the moving target.

Figure 4A shows that under these conditions, the population’s mean protein output (blue points) closely tracks the sinusoidal target (yellow line) throughout the simulation. Because the parameters can shift substantially from one generation to the next, the population can quickly adjust to each crest and trough of the sine wave.In distance–protein space, the population repeatedly shifts in sync with the changing environment.

By contrast, small-step mutations (Figure 4B) produce a “sawtooth” pattern: by the time the population partially aligns with one peak of the sine wave, the environment has already shifted toward the next trough. Consequently, the population consistently lags behind the moving target. The distance–protein plot confirms narrower movement, reflecting insufficient mutation amplitude to handle rapid oscillations.

These findings highlight the importance of mutation amplitude in environments with rapidly shifting demands. When all parameters can undergo large changes, the population readily follows each oscillatory cycle, effectively “chasing” the target in real time. In contrast, small mutation steps produce persistent lags and sawtooth-like deviations from the sine wave.

## Methods

### Gene expression model

We model gene expression as a simplified two-step linear system involving transcription and translation (Miró-Bueno and Rodríguez-Patón, 2011). The dynamics of mRNA and protein molecules are governed by:

(2) $$\frac{d(\text{mRNA})}{dt}=K_{0}F-K_{2}\text{mRNA},$$

(3) $$\frac{dP}{dt}=K_{1}\text{mRNA}-K_{3}P,$$

where mRNA is the number of mRNA molecules, *P* is the number of protein molecules, *K*_0_ is the transcription rate, *K*_1_ is the translation rate, *K*_2_ and *K*_3_ are the mRNA and protein degradation rates, respectively, and *F* is a transcription factor or regulatory signal (held constant in this model).

To analyze long-term behavior, we focus on the **steady state**, where $\frac{d(\text{mRNA})}{dt}=0$ and $\frac{dP}{dt}=0$. Solving these algebraically yields:

(4) $${\text{mRNA}}_{\text{ss}}=\frac{K_{0}F}{K_{2}},\;P_{\text{ss}}=\frac{K_{1}{\text{mRNA}}_{\text{ss}}}{K_{3}}=\frac{K_{0}K_{1}F}{K_{2}K_{3}}$$

Since *F* is constant and fixed across simulations, we absorb it into the target phenotype value, defining:

(5) $$P_{\text{ss}}\propto \frac{K_{0}K_{1}}{K_{2}K_{3}}$$

This steady-state assumption underlies our model’s phenotype definition: any set of parameters (*K*_0_, *K*_1_, *K*_2_, *K*_3_) yields a steady-state protein output *P*_ss_. Notably, multiple parameter sets can converge on the same protein level; thus, phenotype alone does not reveal the exact underlying rates.

### Genetic algorithm

To simulate evolutionary adaptation, we implement a genetic algorithm where each individual is characterized by a 4-tuple (*K*_0_, *K*_1_, *K*_2_, *K*_3_). The algorithm initializes a population of *N* random sets, evaluates each based on its resulting *P*_ss_, and compares that to a target phenotype. High-fitness individuals are selected to reproduce, introducing mutations (small random changes) into their parameter sets. Over successive generations, the population refines its parameters to better match the target.

All the simulations were ran with an initial population of 200 individuals, a mutation rate of 0.1 (probability of mutating each parameter) and were stopped to the target protein when mean generation output was less than 10% of the target. When a parameter is mutated, its value is randomly modified uniformly by 0.5% of its range using a small mutation scale or 5% using large mutation scale. This last large mutation scale was the one used in all of the simulations in this study, except for the case of the time varying target, were we tested both scales.

### Parameter ranges

We define $P=\frac{K_{0}K_{1}}{K_{2}K_{3}}$, with parameter ranges derived from the literature (Bernstein et al., 2002; Proshkin et al., 2010; Gong et al., 2008; Valleriani et al., 2011; Nagar et al., 2021).

To convert experimental half-lives to rate constants, we use:

(6) $$k=\frac{\ln(2)}{t_{1/2}}$$

#### mRNA degradation

Half-lives range from 3 to 8 minutes for most E. coli mRNAs (Bernstein et al., 2002).

#### Protein degradation

Fast-degrading (FD) proteins have half-lives of 2.5–7.5 min; slow-degrading (SD) proteins range from 6–10 min (Nagar et al., 2021).

#### Transcription/Translation rates

Transcription elongation rates range from 12–42 nt/s (Proshkin et al., 2010); mRNA lengths span 300–1800 nt (Valleriani et al., 2011). Translation rates are 4–14 aa/s, with protein lengths from 100–500 aa (Gong et al., 2008).

Tables 1 and 2 summarize these values.

## Discussion & Conclusions

In this study, we explored how variability in gene expression parameters shapes evolutionary trajectories beyond phenotypic outcomes alone. By defining individuals as four-rate models of transcription, translation, and degradation (mRNA and protein), and simulating adaptation in populations of individuals with a genetic algorithm, we showed that identical steady-state protein levels can arise from distinct genotypes. Our key finding is that the *parameter distance*—the summed relative deviation of each rate from a reference set—strongly influences both adaptation speed and the routes taken in parameter space. Populations closer in parameter distance to the optimal configuration generally adapted more rapidly via direct trajectories, whereas those farther away exhibited slower, more circuitous paths. This highlights that evolutionary studies must consider underlying kinetic architectures, not just phenotypic similarity. Although alternative metrics exist to quantify differences between parameter sets—such as cosine similarity (Singhal, 2001), which evaluates the relative proportions between parameters independent of their absolute magnitudes—we chose a parameter distance metric based on normalized absolute differences. This choice reflects our focus on analyzing variations in the actual parameter values themselves, rather than only their relative relationships. However, in future work, exploring alternative distance measures could provide complementary insights. Assessing how such metrics affect the interpretation of parameter divergence and evolutionary trajectories remains an interesting avenue for further investigation.

Moreover, populations with higher sensitivity to mutations adapted more rapidly to changing environments, while those with lower sensitivity exhibited robustness that slowed evolutionary change. These findings align with previous research indicating that the evolvability of gene expression is influenced by factors such as network topology Grozinger and Goñi-Moreno (2023) and the presence of regulatory motifs like TATA boxes, which affect mutational sensitivity so that greater sensitivity to mutations leads to more sensitivity to systematic environmental perturbations (Landry et al., 2007). Recent work by Abreu et al. (2024) further supports the view that environmental history can modulate the fitness effects of mutations: their large-scale lineage tracking in yeast revealed that fitness in fluctuating environments often deviates from a simple time-averaged expectation due to environmental memory, whereby the effects of mutations depend not only on the current condition but also on prior environmental states (Abreu et al., 2024). This suggests that adaptive potential is shaped not only by static mutation effects but also by how those effects are contextually and temporally expressed across environmental shifts. Such insights reinforce the importance of considering not just mutational impact but also the structure and cadence of environmental change when evaluating evolvability.

Designing biological circuits solely for fixed performance can limit their long-term functionality, especially in dynamic environments. Living systems have evolved to process information through mechanisms shaped by mutation, selection, and adaptation, offering a form of computation fundamentally different from that of electronic systems (Grozinger et al., 2019). Recognizing that biological function emerges through evolutionary processes opens the door to circuit designs that are not just robust to change, but capable of improving through it (Grozinger et al., 2025). By incorporating tools like parameter distance into the design process, it may become possible to anticipate how systems might evolve and to intentionally guide this change. This approach turns evolution from a challenge into an opportunity, enabling the development of adaptable, resilient biological technologies.

In summary, by quantifying emerging regulatory variation through parameter distance, we reveal a deeper layer of evolutionary dynamics. Incorporating this perspective into both evolutionary theory and synthetic biology design promises to unlock new strategies for engineering living systems that are not only functional at a single time point but evolvable and robust into the future.

## Figures and Tables

**Table 1:** Degradation parameter values.

Parameter	K_2_	K_3FD_	K_3SD_
min *t*_1/2_ [min]	3.0	2.5	6.0
max *t*_1/2_ [min]	8.0	7.5	10.0
min *K_i_* [min^–1^]	0.087	0.092	0.069
max *K_i_* [min^–1^]	0.231	0.277	0.116

**Table 2:** Transcription/translation parameter values.

Parameter	K_0_	K_1_
min *K_i_*	12 nt/s	4 aa/s
max K*_i_*	42 nt/s	14 aa/s
min length	300 nt	100 aa
max length	1800 nt	500 aa
min *K_i_* [min^–1^]	0.40	0.48
max *K_i_* [min^–1^]	8.40	8.40
